# Historic Timber Roof Structure Reconstruction through Automated Analysis of Point Clouds

**DOI:** 10.3390/jimaging8010010

**Published:** 2022-01-13

**Authors:** Taşkın Özkan, Norbert Pfeifer, Gudrun Styhler-Aydın, Georg Hochreiner, Ulrike Herbig, Marina Döring-Williams

**Affiliations:** 1 Institute of History of Art, Building Archaeoloy and Restoration, Vienna University of Technology, Karlsplatz 13/E251, 1040 Vienna, Austria; gudrun.styhler@tuwien.ac.at (G.S.-A.); ulrike.herbig@tuwien.ac.at (U.H.); doering-williams@tuwien.ac.at (M.D.-W.); 2 Research Unit of Photogrammetry, Department of Geodesy and Geoinformation, Vienna University of Technology, Wiedner Hauptstraße 8/E120, 1040 Vienna, Austria; norbert.pfeifer@tuwien.ac.at; 3 Institute for Mechanics of Materials and Structures (IMWS), Vienna University of Technology, Karlsplatz 13/202, 1040 Vienna, Austria; georg.hochreiner@tuwien.ac.at

**Keywords:** architectural modeling, historic building structures, terrestrial laser scanning, point cloud processing, 3D modeling

## Abstract

We present a set of methods to improve the automation of the parametric 3D modeling of historic roof structures using terrestrial laser scanning (TLS) point clouds. The final product of the TLS point clouds consist of 3D representation of all objects, which were visible during the scanning, including structural elements, wooden walking ways and rails, roof cover and the ground; thus, a new method was applied to detect and exclude the roof cover points. On the interior roof points, a region-growing segmentation-based beam side face searching approach was extended with an additional method that splits complex segments into linear sub-segments. The presented workflow was conducted on an entire historic roof structure. The main target is to increase the automation of the modeling in the context of completeness. The number of manually counted beams served as reference to define a completeness ratio for results of automatically modeling beams. The analysis shows that this approach could increase the quantitative completeness of the full automatically generated 3D model of the roof structure from 29% to 63%.

## 1. Introduction

The professional examination of existing roof structures made of timber represents a separate field of activity for various disciplines. In the field of monument preservation, for example, historical roof structures have to be assessed and classified with regard to their monument value in order to ensure an appropriate future handling of the structures [1]. Historical building research is also dedicated to the development of traditional structures and their (often regional) peculiarities in the field of roof structure research [2]. Planners and structural engineers often have to evaluate historic roof structures in terms of their stability or in relation to necessary retrofitting measures. Another field of work is roof extensions, which are often used as a measure to provide living space, especially in dense urban centers. For all exemplary mentioned fields of work, scaled plans and/or digital models of the existing roof structures are today a basic requirement for further work steps.

The traditional method of surveying roof structures uses tape measures, paper and pencil. It assumes vertical and parallel structures and requires a lot of manual effort. Thus, there is a need to automate this process. Terrestrial laser scanning (TLS) is a method to collect millions of points on object surfaces within a short period of time [3]. Alternatively, structure from motion can be used to generate point clouds [4,5]. Many investigations showed that laser scanning is more precise, but requires a higher investment at the beginning [6,7,8,9].

Reconstruction from laser scanning point clouds, also called reverse engineering, is a technique regularly applied to document entire factories and installations within these factories [10]. There is a concentration on piping installations, and high levels of automation can be reached [11]; however, historic roof structures are different for a number of reasons. Firstly, the cross sections are typically rectangular or polygonal but not circular. Secondly, historic roof structures are characterized by limited regularity, despite their generally repetitive layout. The latter, thirdly, leads to a lot of occlusions, which hampers reconstruction.

This paper suggests a set of methods, which are combined in a workflow, to increase the automation of structural and architectural 3D modeling of historic timber roof structures using TLS point clouds.

### Related Work

Continuous development of 3D data capturing devices and used techniques in the field of measuring accuracy and data collection speed supports the usage of point cloud with the objective of sustainable documentation or parametric modeling. Modeling of 3D data as simple geometric primitives is a way of representing it in a compact manner and simplifies any subsequent analysis that would be performed [12].

Up to present, there were several studies related to retrieving 3D parametric models directly from point clouds. Rabbani and Heuvel [13] developed a method for 3D reconstruction of industrial sites using a combination of images and point clouds focusing on detection of plane and cylinder objects using the Hough transform. Masuda and Tanaka [14] presented a solid modeling methodology based on a reflectance image and mesh models that are based on laser scanner point clouds. Poullis [15] proposed a framework for the automatic modeling from point cloud data. Three-dimensional models are generated based on the roof outlines extracted from airborne laser scanner point clouds. Son et al. [10] assumed industrial instrumentation objects as cylinders or boxes that fit to the acquired set of 3D point clouds. Ochmann et al. [16] presented an automated method for reconstruction of room models from indoor point clouds to transfer the model to Building Information Modeling (BIM) using the Industry Foundation Classes (IFC) standard.

Balletti et al. [17], had a research study on the wooden structure of the dome of SS. Giovanni e Paolo in Venice. Taking the advantage of laser scanning survey, they shortened the data acquisition time on site; however, the final result, a 3D model, was obtained after some months of data elaboration and modeling in Computer-aided desing (CAD) software. The work of Cabaleiro et al. [18] provided a methodology for 3D modeling of highly irregular timber beams from TLS data using cross sections. In this approach, cross sections are assumed to follow a certain pattern, and this external knowledge must be brought in. Pöchtrager et al. [19] presented a highly automated methodology to process point clouds from laser scanning data to 3D modeling of linear beams of historic timber roof structures. In the further study of Pöchtrager et al. [20], the extended version of the developed method was applied on point clouds with different point density or completeness. Even if the method fits well for processing and modeling of entire roof structure point cloud, approximately 30% of beams of a historic roof structure could be modeled without any manual operation, and the aim is to obtain a higher completeness. Murtiyoso and Grusenmeyer [21] were inspired from exhibit [20] in modeling of timber beams. Additionally, they proposed a Hough transform analysis based method for separation of Y- or L-shaped point cloud segments into I-shaped sub-segments. The method was applied to less complex roof structures and solves only a part of the entire task.

## 2. Materials and Methods

### 2.1. Roof Structure Site

To evaluate the suggested methods and the workflow from scratch to end, the roof structure of St. Michael, a medieval church in the city center of Vienna, was chosen as a case study. As part of the medieval Vienna Imperial Palace, the roof structures of the church have been analyzed for several years by experts of the Vienna University of Technology in cooperation with the Federal Monument Authority of Austria and further scientific partners in the frame of research-based student courses [22]. The selected roof structure of St. Michael was newly erected after 1525, when a disastrous city fire destroyed the former structure [23]. It was built as a rafter roof with a double standing truss combined with hanging struts in the ridge level and represents a typical construction of late medieval carpentry.

**Figure 1:**
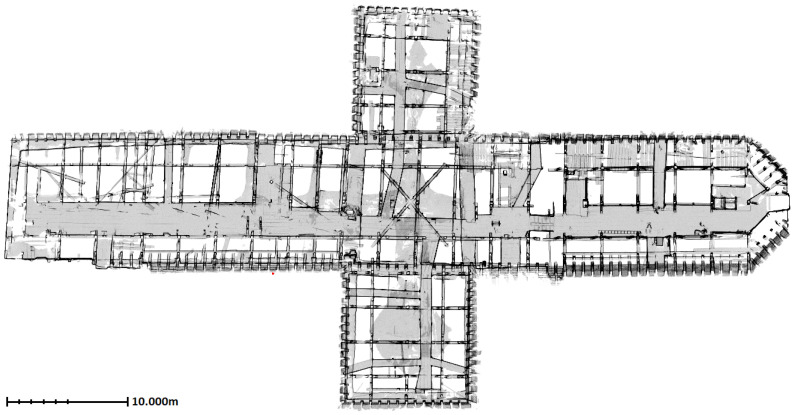
The ground plan obtained from point cloud of the roof structure of St. Michael.

For a visual impression, the ground plan of the roof structure is presented in Figure 1. In perspective of dimensions, the length of the roof structure along the longitudinal axis above nave and chancel is around 65 m, the extend of the latitudinal axis above the transept is 32 m, height is near to 10.5 m and the span length of the vertical slice of the repetitive sections is nearly 11.5 m. Distance between neighbor rafters is approximately 70 cm. The roof structure consists of around 1 m to 10 m long timber beams with the width of 10 cm to 25 cm. As the roof structure comprise two floors, there are also wooden walking ways and railings located on both floors. Stairs, infrastructure elements (cables, etc.) and roof covering are some other visible objects that are irrelevant for architectural or structural modeling.

### 2.2. Point Cloud Acquisition

Different sensors and techniques can be used to acquire 3D point cloud data. These methods include 3D laser scanning, photogrammetry, videogrammetry, RGB-D camera and stereo camera [24]. TLS have a great potential due to their ability to capture objects in high speed with accuracy up to sub-millimeter [25]. The capability of obtaining highly detailed and accurate point cloud within minutes is fitting well with surveying and documentation of structures such as historic timber roofs. The principle result of single scanning is a 3D point cloud in the scanner coordinate system. To cover as much as possible surfaces of the working area, scanning needs to be applied from more than one position. Afterwards, point clouds from different scan positions can be transformed into a superior coordinate system using artificial targets or a registration approach such as Iterative Closest Point (ICP) [26].

Scanning of the roof structure was made with a Riegl VZ-2000i [27] device. The device has the ability of up to 500,000 points measurement per second, 3 mm to 5 mm accuracy within a range of 1 m to 2.5 km. The scanning was planned to cover as much as possible of the dense structure of the roof. Therefore, in both floors the scanner was positioned between rafters sequentially keeping approximately 2 m distances. Totally, 241 scans were made and each scan took around 20–40 s. Over 600 million points from inside the roof structure were collected at the end of one day of work. While the coarse registration of the scans was already performed internally by the scanner for spatially consecutive scan positions, coarse registration of non-registered scan positions, refinement of the registration and multi-station adjustment applications were performed afterwards using RiScanPro [28] software. While the registration after scanning was completed within two working days, the multi-station adjustment process resulted in 0.004 m root mean square error (RMSE) in approximately 40 min.

### 2.3. Method

#### 2.3.1. Overview

The methods and workflow applied in this study aim to reduce the manual effort during parametric 3D modeling of historic timber roof structures using TLS point cloud data. The process chain takes registered point clouds and corresponding scan positions as input data. After the registration, the point clouds can be exported separately for each scan position or as a single file, which is a combination of selected scans. To determine the beam surface normal directions, the relation between each point and the source scan position are used during normal vector computation.

In this study, timber beams of the roof structure are assumed as straight or slightly bending objects with rectangular cross shapes. As a result of that, the primary output is a set of cuboid objects, which include dimensions, orientation and position for each timber beam within the same coordinate system as the point cloud. Woodworking joints are detected and stored with the help of intersections of cuboid objects.

**Figure 2:**
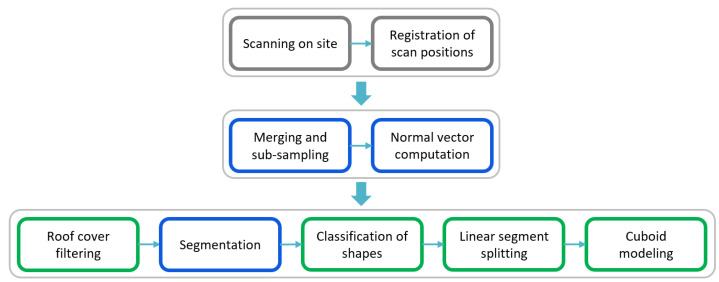
Main stages of the 3D modeling workflow.

Figure 2 shows the main workflow of this study. The gray colored steps in the first stage cover on site work and registration of multiple scan positions. Second stage covers general point cloud pre-processing operations. The third stage is specific to the historic roof structures. Blue colored steps are applied with OPALS [29] software, a point cloud processing software and the resulting point cloud and computed attributes are stored in the OPALS Datamanager (ODM) [30] file. All the algorithms and process chains shown in green colors are developed in Python [31]. In the last stage, while classification of shapes and cuboid modeling steps are applied with the same approach as in the Pöchtrager et al. (2017, 2018) method, new roof cover filtering and linear segment splitting steps are developed and included to the workflow for the purpose of both data simplification and increased automation.

In the first new method presented in this article, roof cover filtering is performed, in order to avoid processing irrelevant points. The point cloud is separated into the roof face and the roof interior. The roof face consists of large planes, whereas the interior includes the roof structure, our primary object of interest, and other elements such as infrastructure (cables) and scanning artefacts (persons). The interior is segmented into planar or slightly bending objects in the next step. In the classification step, segmentation results are grouped into linear, non-linear separable and complex segments. Linear segment splitting, the second new method in this workflow, is developed to split the non-linear separable segments into linear sub-segments. Finally, cuboid fitting is performed using all linear segments after which woodworking joints are detected using cuboid intersections.

#### 2.3.2. Combining of Point Clouds

Manufacturers of TLS devices provide their own software to fulfill the need of point cloud processing operations such as registration, adjustment and data export. Registration of scan positions and multi-station adjustment of the scans are applied on RiScanPRO [28], which is the software provided by RIEGL, (Horn, Austria). While the registered point clouds from different scan positions are exported as separated files in the .las [32] format, the final positions of each scanner, more precisely the origin of the scan in the superior coordinate system, are available (as a text file) as well.

A single point cloud file can be generated using separated .las files and the corresponding scan positions. In the merged file all points from all scan positions are stored, augmented by information of the measurement rays. They are also stored for each point using Equation (1).

(1) $$\vec{Ray_{i}}=\left(P_{i}-S_{P_{i}}\right)$$

In Equation (1), $P_{i}$ are the coordinates of point i measured by scan position $S_{Pi}$ and $\vec{{Ray}_{i}}$ is direction vector of point i.

As the scanning is applied from different positions inside the roof structure, the merged point cloud of all scan positions includes a higher amount of points than is necessary for the next stages of the processing chain. While some regions are visible from multiple scan positions, some others do not contain any points because of the shadow effect. The distance from scanner is also influencing the density of measured points on the surfaces. Sub-sampling is applied to achieve a point cloud with a regular or at least more uniform point density. Pöchtrager et al. [20] pointed out that lower point density had only little effect on the quality of final model. During sub-sampling, only one point within a sphere defined with a search radius (e.g., 0.01 m) is stored while other points are ignored. As a result, sub-sampled points are measured real (not interpolated) scan points. A robust plane is fitted to *k*-nearest neighbor (e.g., k=16) inside a search sphere to compute normal vectors of each point. Computed normal vectors are oriented towards the scanner using ray vectors (Equation (1)). The normal vectors are oriented outwards, which is obtained by ensuring that the normal vector and the ray vector for each point have a negative dot product.

#### 2.3.3. Roof Cover Filtering

**Figure 3:**
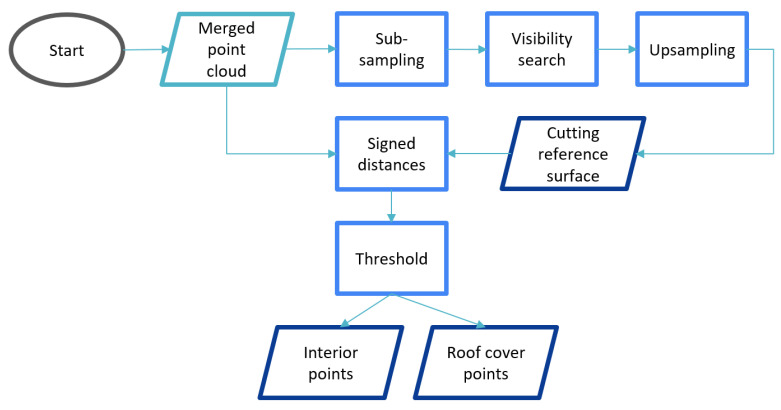
Workflow of roof cover filtering.

The roof cover filtering is a set of automated steps, which allows to separates the point cloud into exterior and interior points (Figure 3). This does not only exclude roof faces but also the ground from the entire point cloud.

The filtering has two sub-steps: (i) the preparation of the cutting reference surface; (ii) the comparison of the reference surface and the source point cloud.

##### Preparation of Cutting Reference Surface

As the roof structure is scanned from the inside, the point cloud includes roof cover surfaces as well as timber beam, wooden walking ways, etc. From an outer view point, the entire point cloud is covered by mostly roof material. Interior and exterior points are separated by a reference surface, which surrounds the entire point cloud from outside.

Even if the surrounding surface mostly contains roof material and ground points (located on the bottom of the point cloud), there are also points of structural elements that have continuous contact with the roof cover. A voxel-based sub-sampling is applied to the entire point cloud with a search radius approximately equal to the maximum width of beams directly connected to the roof cover (e.g., 15–20 cm) in order to reduce the number of the points belonging to these beams.

**Figure 4:**
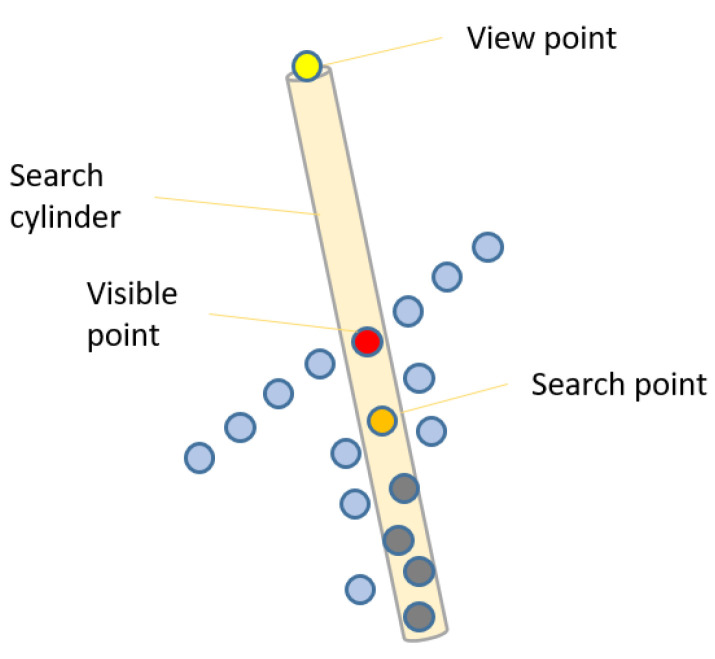
Hidden point removal approach.

Generation of a reference surface that is fitting well to the roof cover is achieved through application of a hidden point removal [33] method. First, the Euclidean distance between a point and the input view position is stored as attribute. Then, the points that have minimum distance value within an oriented infinite cylinder search region (e.g., radius = 10 cm) are considered as visible points as shown in Figure 4.

Figure 4 illustrates a cylinder, a search point (orange), a view point (yellow), and the visible point that has minimum distance to view point (red). Points that have larger distance value (gray) are not considered further, until the view point is changed, in order to improve the runtime performance.

Artificial view points are defined for top, bottom and four sides. The hidden point removal method for the six points of view provides output points, which cover the object from outside where these points are stored as a single point cloud.

(2) $$k=\sqrt{{\left(X_{Max}-X_{Min}\right)}^{2}+{\left(Y_{Max}-Y_{Min}\right)}^{2}+{\left(Z_{Max}-Z_{Min}\right)}^{2}}$$

(3) $$\begin{array}{c} V_{1}=\left(X_{Center},Y_{\mathrm{Center}},Z_{\mathrm{Max}}+k\right)\\ V_{2}=\left(X_{\mathrm{Center}},Y_{\mathrm{Center}},Z_{\mathrm{Min}}-k\right)\\ V_{3}=\left(X_{\mathrm{Max}}+k,Y_{\mathrm{Center}},Z_{\mathrm{Center}}\right)\\ V_{4}=\left(X_{\mathrm{Min}}-k,Y_{\mathrm{Center}},Z_{\mathrm{Center}}\right)\\ V_{5}=\left(X_{\mathrm{Center}},Y_{\mathrm{Max}}+k,Z_{\mathrm{Center}}\right)\\ V_{6}=\left(X_{\mathrm{Center}},Y_{\mathrm{Min}}-k,Z_{\mathrm{Center}}\right) \end{array}$$

In Equation (3), $V_{1\ldots 6}$ are the selected view positions, *k* value in Equation (2) is the maximum distance between bounding box vertices, (X,Y,Z)_Center_ is middle point of the bounding box of the input point cloud, (X,Y,Z)_Min_ and (X,Y,Z)_Max_ are the limits of the input point cloud.

Although the combination of all the results from hidden point removal applied with $V_{1\ldots 6}$ point clouds represents the exterior surface of the entire point cloud, it still does not have the same density as the original input point cloud. For a reliable comparison of two point clouds, surface reference point cloud needs to be up-sampled as similar as the input point cloud. For this reason, Moving Least-Squares (MLS) [34] method is applied to the point cloud to up-sample it to a higher density value (e.g., 0.01 m).

##### Point Cloud Split into Roof and Interior

The next step is to compute signed distances between the cutting reference surface point cloud and the input point cloud. At the beginning, normal vectors of the cutting reference surface point cloud are oriented from center to the outer side of the point cloud. Then, the signed distances between the input point cloud and cutting reference surface are computed with the Equation (4).

(4) $$\begin{array}{c} d_{\mathrm{signed}}=\vec{n_{B}}\cdot \left(\vec{P_{A}-P_{B}}\right) \end{array}$$

In regards to Equation (4), $P_{A}$ is the point from input point cloud, $P_{B}$ is the closest point to $P_{A}$ from cutting reference surface point cloud and $\vec{n_{B}}$ is the normal vector of the point $P_{B}$.

While the negative *d*_signed_ values mostly represent the roof structure interior, positive values mean that the points are outside of the cutting reference surface point cloud. With the signed distance information, a threshold value *t*_distance_ can be specified taking the roof cover material thickness into account to separate the interior part. Usage of the threshold value may exclude some of the beam side face points that are close to the roof cover. This can be avoided by additionally considering the angles between the normal vectors.

**Figure 5:**
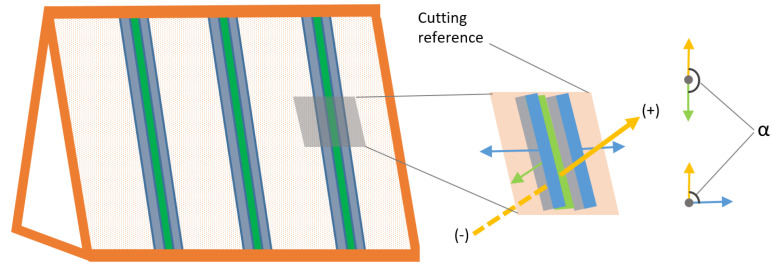
Side beam faces cut by reference surface (light-blue), interior side face regions (gray) and equivalent orthogonal face (green).

As visualized in the Figure 5, the light-blue colored regions are assumed as exterior regions because of the *t*_distance_ value. To achieve this, α, angle between normal vectors of cutting reference point cloud and input point cloud (Equation (5)), is used for an additional thresholding in Equation (6). The α is visually shown in Figure 5 (right) for both green and blue regions. The points fulfilling Equation (6) are the interior roof point cloud.

(5) $$\alpha =\arccos(\vec{n_{\mathrm{ref}}}\cdot \vec{n_{\mathrm{input}}})$$

(6) $$d_{\mathrm{signed}}<t_{\mathrm{distance}}\vee \left(d_{\mathrm{signed}}\geq t_{\mathrm{distance}}\wedge \alpha >t_{\mathrm{angle}}\right)$$

In Figure 5, blue and green arrows are normal vectors of beam side faces, while yellow arrow is the normal vector of the cutting reference surface. The blue side faces are physically connected and almost perpendicular to the cutting reference surface, whereas the green face has no connection. The specified $t_{\mathrm{distance}}$ (e.g., 5 cm) and $t_{\mathrm{angle}}$ (e.g., 85°) values inside Equation (6) can separate interior points including light blue regions shown in Figure 5.

#### 2.3.4. Segmentation

Segmentation is used to detect planar side faces of beams in the point cloud. These side faces will be used in a later stage for fitting cuboids to the point clouds of adjacent beam sides. The interior roof point cloud (previous section) is the input for segmentation. Segmentation consist of following steps;

- Region-growing-based segmentation.
- Planar sub-segmenting.

The angle between normal vectors within a local neighborhood (e.g., *r*_max_ = 0.05 m) is used as homogeneity rule for a region-growing-based segmentation.

(7) $$\arccos\left(\vec{n_{p}}\cdot \vec{n_{n}}\right)<\alpha_{\max}$$

In Equation (7), $\vec{n_{p}}$ and $\vec{n_{n}}$ are unit normal vectors of a point and its candidate neighbor while α_max_ is a threshold value for local angular homogeneity (e.g., 5°).

Related to the specified alpha_max_ value, segmentation result may contain not only planar segments but also non-planar ones. The least squares fitting plane [35] is calculated for all segment points. The root mean square error (RMSE) of plane fitting is the reference value for decision of searching for planar sub-segments. If the plane is not fitting well to all segment points (e.g., RMSE > 0.04 m), the RANSAC algorithm [36] is used to detect multiple planar sub-segments as explained in [19,20].

#### 2.3.5. Shape Classification

**Figure 6:**
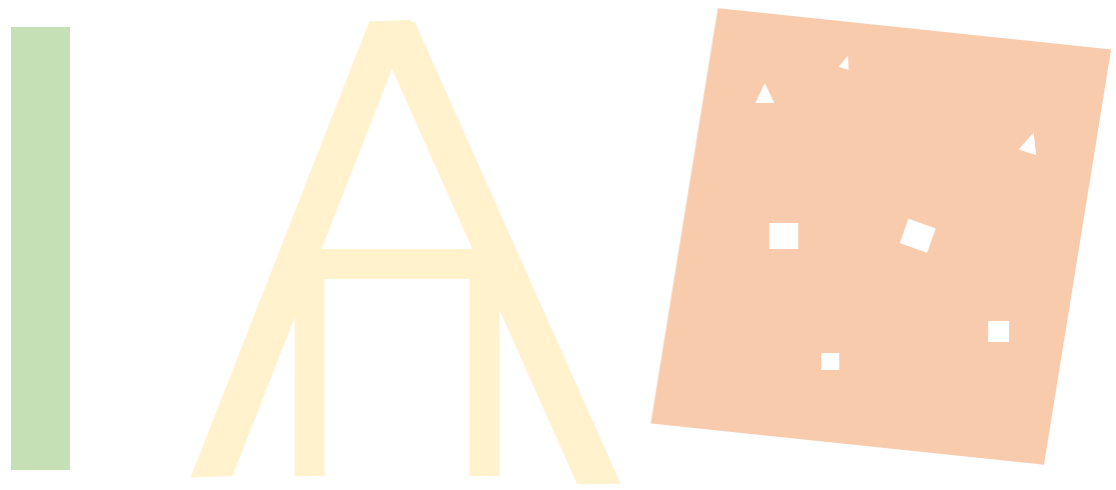
Type-1 (**left**), Type-2 (**center**) and Type-3 (**right**) segments visualization.

The results of the segmentation comprise planar point sets, which do not only contain single beam side faces, but also other objects such as walls or wooden walking ways. To define the segments that belong to the beam side faces, main input for the cuboid fitting, the segmentation result needs to be classified. The aim of this step is to classify segments into following three types (Figure 6) using shape factors computed with Equation (8):

- Type-1: Linear shaped segments.
- Type-2: Non-linear segments with separable sub-segments.
- Type-3: Non-linear compact segments.

(8) $$\begin{array}{c} f_{\mathrm{elong}}=\sqrt{\frac{\lambda_{l}}{\lambda_{w}}}\\ f_{\mathrm{area}}=\frac{A_{\alpha }}{A_{\mathrm{MBR}}} \end{array}$$

Regarding to Equation (8), λ_l_ and λ_w_, “length” and “width” of a segment, are the largest and second-largest eigenvalues, which are computed for all segment points with principle component analysis [37], A_α_ and A_MBR_ are the area of the alpha-shape [38] and its minimum bounding rectangle (MBR) for a segment. In conclusion, for a segment, if $f_{\mathrm{elong}}$ > 5 and $f_{\mathrm{area}}$ > 0.5 then it refers to type-1, if $f_{\mathrm{elong}}$ < 4.5 and $f_{\mathrm{area}}$ > 0.8 then it corresponds to type-3, while all other cases considered type-2 [20].

**Figure 7:**
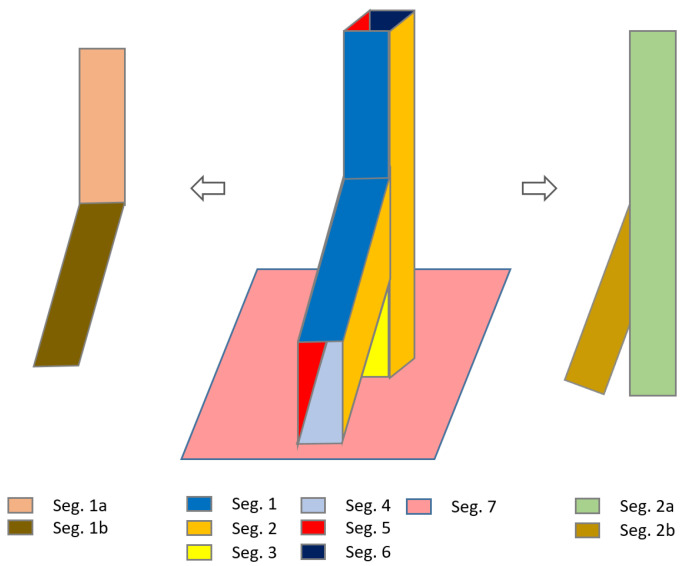
Region growing segmentation result (in the **middle**), planar sub-segmentation with RANSAC (**left**) and non-linear segment with separable sub-segments (**right**).

Figure 7 shows that Segments 3, 4 and 6 are linear segments named as type-1, which are candidates to be a side face of a timber beam. Segment 1 needs to be separated to planar sub-segments while Segment 2, classified as type-2, has separable linear sub-segments (2a and 2b) and Segment 7 is a compact segment that is ignored in the further process.

#### 2.3.6. Linear Shaped Segment Splitting

The segments classified as type-2 need to be split into linear sub-segments (2a and 2b) as shown in Figure 7, in order to be involved in cuboid fitting. Cuboid modeling results of Pöchtrager et al. [20] show that the number of modeled beams are directly related to the number of detected beam side faces (type-1). Murtiyoso and Grussenmeyer [21] applied a Hough transform analysis after projection of Y or L shaped of type-2 segment points to a binary image to find more beam faces.

**Figure 8:**
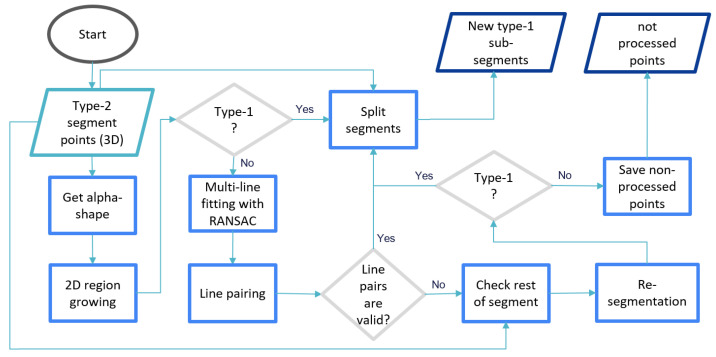
Automation flowchart of linear (straight) part splitting for type-2 segments.

A new method is suggested (Figure 8) which aims at both, increasing of automation and simplifying manual operations during type-2 segment splitting.

Type-2 segment points (3D) are the input of the processing chain. The methods inside the processing chain are then applied on alpha-shape of 2D projection of input 3D points to the best fitting plane that was already computed in previous stage. A 2D region growing segmentation method is applied on the alpha-shape border points at the beginning because complexity and number of sub-segments differs for individual input segments.

(9) $$\mathrm{linearity}=\sqrt{1-\frac{\lambda_{l}}{\lambda_{w}}}$$

The 2D segmentation reduces the complexity by separating parts as large as possible. For this reason the segmentation is applied on 2D alpha-shape vertices that have high linearity value, computed as Equation (9), (e.g., linearity > 0.95). The defined search radius in the segmentation considers the maximum thickness of the beams (e.g., 25 cm) in order to detect as large as possible straight line segments.

The result of 2D segmentation may contain both type-1 and type-2 shaped segments. After checking the types using shape factors 8, while type-1 labeled parts are candidates to be a side face, type-2 labeled ones need further processing. An iterative line fitting (using RANSAC algorithm) is performed on type-2 labeled segments in the second stage.

In Equations (10), the resulting lines defined with $m_{i}$, $b_{i}$ are compared to each other and $\alpha_{i,j}$ is computed as an angle between the two lines. If the angle between line i and line j is less than specified in the threshold (e.g., 5°), then line j is a candidate to be a match with line i.

(10) $$\begin{array}{c} y=m_{i}x+b_{i}\\ \alpha_{i,j}=\arctan\left(\frac{m_{i}-m_{j}}{1-m_{i}\cdot m_{j}}\right) \end{array}$$

Euclidean distances are computed from the center point of reference line segment to all candidate lines. If there are more than two candidates within the expected beam width (e.g., 15–30 cm), one out of the two closest candidates is the correct match. The reference line is paired with both candidates for checking and then computing convex-hull geometry (using vertices of reference and candidate lines) intersected with alpha-shape geometry of the input segment. The correct match should have higher intersection area ratio. If shape factors and intersection ratio supports the sub-segment, a new segment ID is assigned to the points inside the convex-hull polygon. All other points are neglected. When the last sub-segment candidate is checked, an additional segmentation is applied only on non-processed points if the number of points is larger than a threshold value (e.g., 600 points). The last segmentation result is checked directly for type-1 segments to assign the points to a new sub-segment. Finally, all sub-segment points and non-processed points are stored into point cloud. While sub-segments are going to be considered as candidate to be beam side face, non-processed points will have a chance to be partitioned manually.

#### 2.3.7. Cuboid Fitting and Modeling

The final product of the study, parametric models of the beams, is a set of cuboid objects defined with position, dimensions (width, height, length) and orientation information. The cuboid fitting method explained by Pöchtrager et al. [20], extracts all parameters of the cuboid objects if at least two adjacent beam faces are known. The following methods were applied directly as explained in [20]:

1. Identification of adjacent beam segments.
2. Fit cuboids for beams.
3. Intersect beams and analyze the structure.

To define the adjacent segments that form together a beam, between a reference and a candidate segment, the following three conditions need to be fulfilled:

- Distance of centroid of reference to plane of candidate:
  (11) $$\left|C_{A}-P_{B}\right|<dist_{\max}$$
- Angle between normal vectors:
  (12) $$\arccos\left(\vec{n_{A}}\cdot \vec{n_{B}}\right)\approx \left[0,90,180,270\right]$$
- Angle between longitudinal axes:
  (13) $$\arccos\left(\vec{l_{A}}\cdot \vec{l_{B}}\right)\approx \left[0,180\right]$$

A least squares estimation approach is applied for fitting cuboids to the adjacent segment points. The following equations are considered in order to minimize the distance between a side face of cuboid and the point that belongs to the side face:

(14) $$\begin{array}{cc} \; & f1\left(p_{1,j}\right):{\left(p_{1,j}-p_{0}\right)}^{T}\cdot r_{1}=d_{1,j}\\ \; & f2\left(p_{2,j}\right):{\left(p_{2,j}-p_{0}\right)}^{T}\cdot r_{2}=d_{2,j}\\ \; & f3\left(p_{3,j}\right):{\left(p_{2,j}-\left(p_{0}+a\cdot r_{1}\right)\right)}^{T}\cdot r_{1}=d_{3,j}\\ \; & f4\left(p_{4,j}\right):{\left(p_{4,j}-\left(p_{0}+b\cdot r_{2}\right)\right)}^{T}\cdot r_{2}=d_{4,j}\\ \; & p_{0}=CoG+s\cdot r_{1}+t\cdot r_{2} \end{array}$$

In Equations (14), $p_{i,j}$ is point j of side face i (i = 1, 2, 3, 4), $d_{i,j}$ distance of point j to its side face i, $r_{1}$ and $r_{2}$ are the axes of the beam coordinate system, *a* and *b* are beam dimensions in $r_{1}$ and $r_{2}$ direction, CoG is center of gravity of all beam points, $p_{0}$ is the base point of the beam, *s* and *t* are shift of $p_{0}$ from CoG to inner side of the beam in $r_{1}$ and $r_{2}$ directions [20].

A fitting cuboid can be achieved with Equation (14) if at least two adjacent beam faces exist.

Detection and classification of woodworking joints is based on spatial proximity. Joints are located at the intersection of cuboids and stored as a line segment that has shortest distance between beam axes. Cuboid objects and joints can be transformed into .dxf [39] and .step [40] data exchange formats for further investigation and processing in 3D computer-aided-design (CAD) or structural analysis software.

## 3. Results

As mentioned before, the methods were applied to a case study, a late medieval timber roof structure from the first half of the 16th century. In Section 2.1, it was pointed out that the scanner was positioned at approximately every second rafter. This pattern was followed for both first and second accessible floors of the roof structure. The accumulation of the collected points from different scan positions resulted in 634,065,068 points. A part of workflow, beginning from sub-sampling until segmentation stage, gradually decimates the number of points for each stage.

Table 1 shows how many points exist before each process and how many of them are transferred to the next step. While sub-sampling mainly aims to remove spatially closed or duplicated points, roof cover filtering directly affects the points on the roof cover. The segmentation process not only generates segments, but also reduces the number of segmented points ignoring segments that has less points than the constraint of minimum number of points.

**Figure 9:**
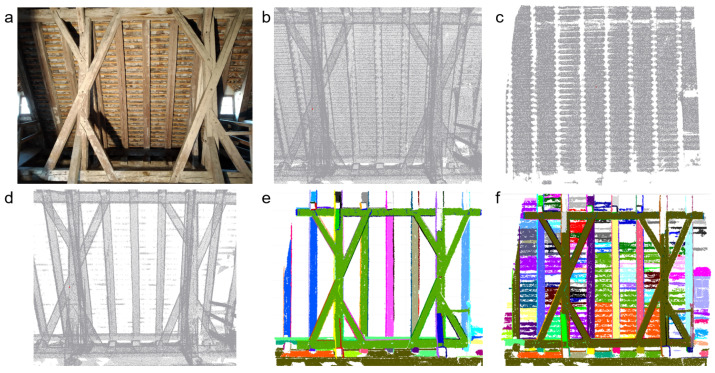
Image of a roof cover part from inside (**a**), point cloud data of the region (**b**), filtered roof cover (**c**), interior points after roof cover filtering (**d**), segmentation result of roof interior after roof cover filtered (**e**) and segmentation result of the point cloud including roof cover (**f**).

The results in Figure 9 show that roof cover filtering was able to separate the most of the roof cover points from point cloud. After the roof cover filtering, the visible small parts belonging to the roof cover in Figure 9d were eliminated during segmentation.

The segmentation step resulted in 5165 segments, of which 5044 are planar and 121 of non-planar segments. While planar segments were directly classified in the next step, non-planar segments are split into planar sub-segments with RANSAC before their classification.

Regarding Table 2, the shape classification ends with 3325 linear shaped segments (type-1), 2021 non-linear segments with separable sub-segments (type-2) and 79 compact segments (type-3).

The 2021 type-2 segments were processed with the newly developed method explained in Section 2.3.6. The method generated 1433 type-1 and 1613 type-2 segments. Thus, 1433 more segments were input data for cuboid fitting and modeling, while 1613 non-linear segments cannot be input data for modeling unless manually partitioned to linear sub-segments.

**Figure 10:**
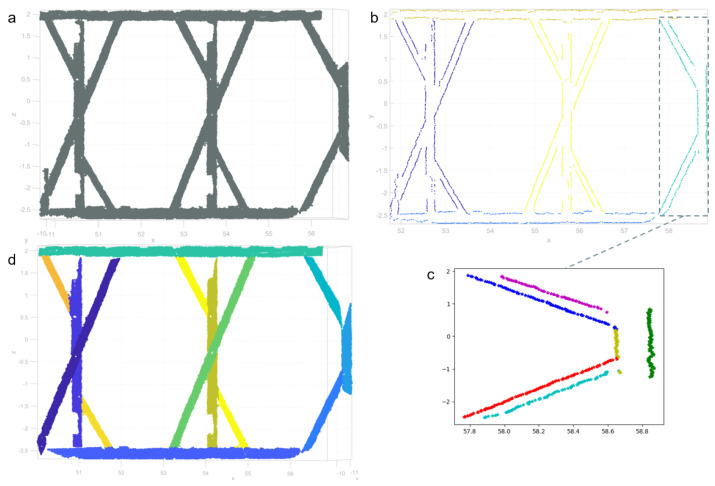
Type-2 segment (**a**), 2D region growing segmentation on alpha-shape (**b**), multi-line fitting with RANSAC (**c**) and linear split sub-segments (**d**).

In Figure 10b, 2D alpha-shape of input type-2 segment (Figure 10a) separated five sub-segments including type-1 (top and bottom) and type-2 (in the middle, magenta, yellow and green colored). Figure 10c shows fitted lines using RANSAC with different colors. Yellow and green, red and turquoise and blue and magenta line pairs are detected as explained in Section 2.3.6. The result of the workflow (Figure 8) presented in Figure 10d shows each linear sub-segments with different colors.

**Figure 11:**
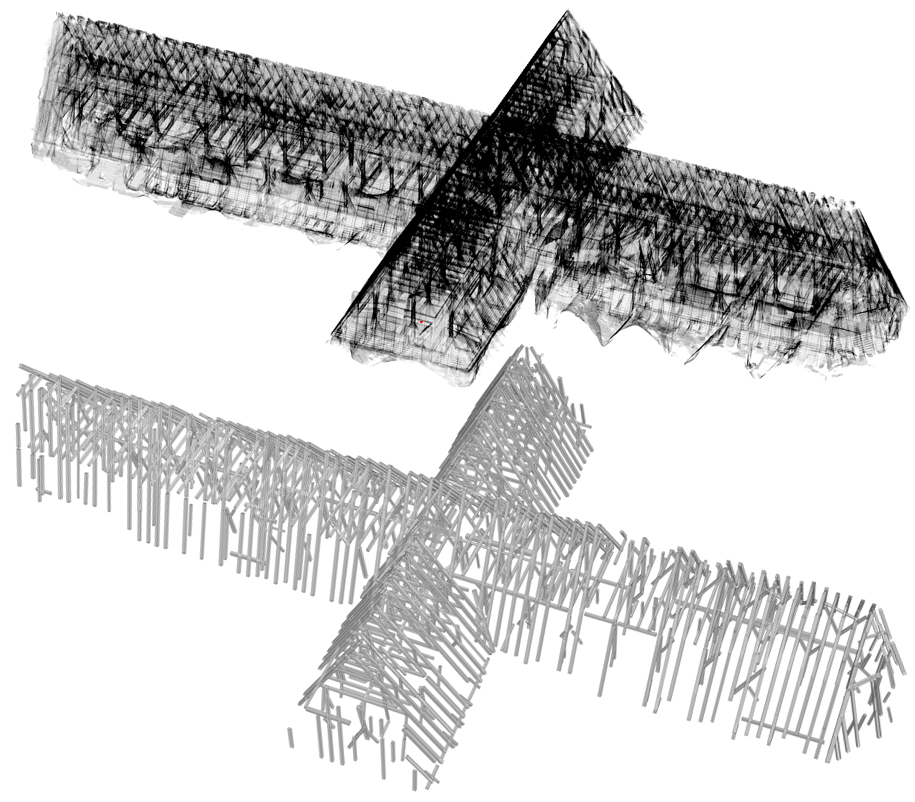
X-ray view of entire point cloud (**top**) and fully automated cuboid fitted beams (**bottom**).

The cuboid fitting was performed on type-1 segments which cover results of shape segmentation (3325) and linear shaped segment splitting (1433). The modeling resulted in 1179 cuboid beams (Figure 11) and 975 joints retrieved by 4758 linear beam side face segments.

As pointed out in Section 2.2, scanning on the site and registration of scan positions took approximately 3 working days, which comprised of 1 day on site study and 2 days of human-interacted work on the computer. The result of the first stage is shown in Figure 11 (top). The following stages of the workflow (Figure 2) were performed fully automatically on a computer using AMD Ryzen 7 2700X (8 × 3.70 GHz) processor and 32 GB RAM capacity. The second stage (subsampling and normal vector computation) completed in 24′01″. The last stage of the workflow that begins from the roof cover filtering to the end of cuboid modeling was performed in 2^*h*^52′52″. The final result (cuboid model) of the main workflow for the entire roof structure of St. Michael is presented in Figure 11 (bottom).

## 4. Discussion

Figure 9e,f highlights that roof cover filtering was able to remove several irrelevant segments for the roof structure modeling. The segmentation step was experienced for both cases (with and without roof cover filtering) with following parameters: *r*_max_ = 0.05 m, α_max_ = $5^{\circ }$ and 600 minimum segment points. The segmentation process resulted in 5533 segments when roof cover is included, while roof cover filtered data resulted with 5165 segments. Thus, roof cover filtering reduced the number of segments by 7%, while the number of points was reduced by 28%. Furthermore, roof cover segments may have shapes similar to beam faces, and thus their removal does not only reduce computation time in later stages but also avoids building beams which are, wrongly, composed of beam faces and roof cover segments.

Method developed by Pöchtrager et al. [20] (Method 1), method presented in this paper (Method 2) and addition of manually split segments inside Method 2 (Method 3) are compared in the following Table 3.

In Table 3 the number-of-beams column was filled manually counting beam elements in the selected regions of the point cloud. For the method columns, fully or partly modeled beams are counted. According to the table, Method 1 is able to extract 29% of existing beams, Method 2, still fully automatically, can increase the ratio of completeness up to 63%. It doubles the number of modeled beams. Method 3 is comprised of Method 2 and additional manually split segments with 1^*h*^45′ manual work duration for both regions. Method 3 is results with 75% of beams modeled.

**Figure 12:**
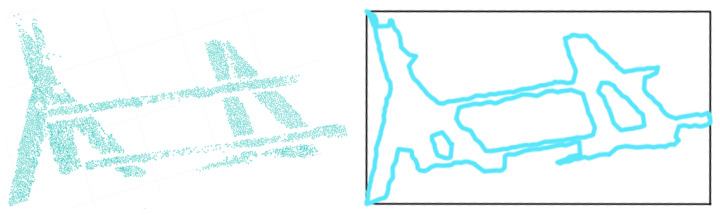
An example of type-2 segment points that cannot be split automatically (**left**), the alpha-shape (turquoise) and minimum bounding box (black) of the segment (**right**).

As presented in Section 3, the automation of type-2 segment splitting generated 1433 of type-1 and 1613 of type-2 sub-segments retrieved from 2021 type-2 input segments. One of the type-2 segments that cannot be split automatically is shown in Figure 12 together with segment points (left) and the alpha-shape geometry (right). The variance of point density and irregularity make the standardization of alpha-shape detection more difficult. While alpha-shape of the segment in the example shown in Figure 10 enables the algorithm to find and pair the straight border lines, because of the irregularity of the alpha-shape in Figure 12, the algorithm is not able to find and match parallel linear sides of linear beam side faces. An additional manual splitting was applied (Method 3) in order to involve linear sub-segments of these type-2 segments into the modeling. The manual operation was finalized in 5^*h*^18′ for the entire roof structure.

Very large point clouds, as the one processed in this example, can be viewed under the aspect of intelligently filtering big data [41]. In this sense the point clouds have large volume ($6.3\times 10^{8}$ points, see Section 3), high velocity of data acquisition (Section 2.2), while veracity may be considered high from the acquisition principle (reliable and precise measurements) but limited for the object to be modeled (relevant and irrelevant points after processing steps in Table 1). A strategy to handle this data, which was also employed in this paper, is the “intelligent filtering” to reduce the number of points (presented in Table 1), to reduce the complexity of the content (roof cover filtering, minimum segment size, removal of type-3 segments), and to reduce computation time as well as to enable manual processing. For the latter it is important that relevant data, i.e., sufficiently generalized data (outlines of alpha-shapes of segments instead of raw points) is generated for the interactive processing.

**Figure 13:**
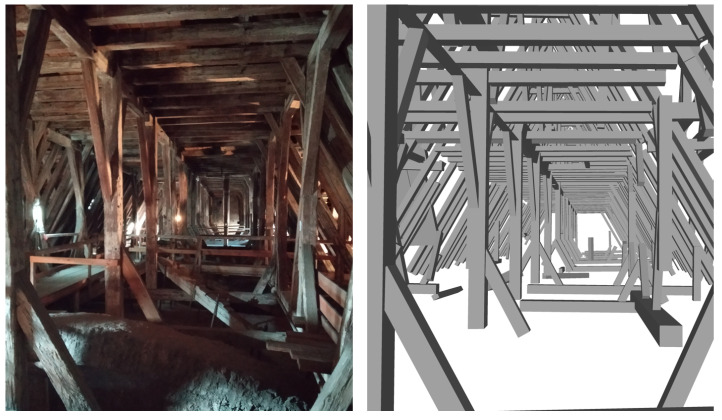
Roof interior image (**left**) and cuboid modeling result of Method 2 (**right**).

Figure 13 presents both an image of the roof interior and the 3D model of the roof structure from the same view point. As clearly seen in the 3D model, non-beam faces that were found during segmentation (e.g., the hand rail or the floor) were not considered during modeling thanks to the applied valid beam width threshold before cuboid fitting (15–30 cm). As seen in the right side of the model, there are some beams, which were modeled partly, which causes insufficient data to form connections between beams. The condition inside the roof structure does not allow the scanning of every single detail with a high point density. Further, the closeness of the rafters causes shadow effects on scanning result. A perfect point cloud without shadows would, of course, increase automation; however, both restrictions in scanning time, and in the possibilities to place the scanner and consider the safety of survey personnel, will not allow us—according to our experiences—to obtain a shadow free point cloud. This causes beam side face segments, thereby the cuboid models not to be extracted properly as a full sized beam. Automatic completion of beams and missing joints and uniform modeling of entire structure is a continuing research topic. It has to be noted that historic roof structures are only semi-regular, and thus a completion based on regularity assumptions should be avoided. Further, for structural modeling, failures are likely to arise where the regularity and continuity are broken. Displacements due to external loading, creep or damage over time make the job of reconstruction of the originally straight beam axis without bending and therefore more perfect structure much more complicated. Pöchtrager et al. [20] suggest manual extension of the beams. For the final model, even if a distinct improvement on automation was achieved, approximately 37% of the beams inside the roof structure cannot be modeled fully automatically. On the other hand, in the final model, there are still several incompletely modeled beams that need further processing. Nevertheless, a significant part of the roof structure could be modeled within a few days after scanning on site.

## 5. Conclusions

The main workflow has been applied on an entire historic roof structure using TLS point cloud. Previous section shows that just 3.62% of collected points is sufficient for 3D model generation of the roof structure. The reduced point cloud not only decreased the processing time for the further steps but also influenced positively additional manual modeling or refinements. Newly applied method that are explained in Section 2.3.6 doubled the number of modeled beams in the final model (Table 3) in comparison with the method presented by Pöchtrager et al. [20]. In the final product, 3D parametric model, a higher degree of automation was reached. A key factor for the completeness is the coverage with points to overcome the occlusions, which are likely to occur in complex roof structures. Furthermore, the factors such as unsuitable conditions on scanning, irregular beam surfaces, deformations or damages on the beams, rigid obstacles in front of the structure elements, etc., bring limitations to the automation. Overcoming these limitations, exploiting the fuzzy or semi-regular structure and bringing in artificial assumption of the missing parts should be considered for an increased automation, but with a clear indication for each model element, if it is based on data or on assumptions, and how strong these assumptions are. The final result is primarily geometry linked to architectural analysis, but will have to be forwarded to engineering aspects such as structural modeling. During the upcoming improvements, the tradeoff between scanning time and completeness of the automatic reconstruction needs to be optimally balanced. As a future aim of this study, efficiency of creating the complete model should be increased, which may include more sophisticated algorithms but also smarter human–computer interaction. Finally, the types of joints between beams should be detected, which requires, however, more input than only the point cloud.

## Figures and Tables

**Table 1:** Number of processed points in context of decimation.

Process Name	Number of Points		Percentage (%)
	Before	After	
Laser scanning	-	634,065,068	100
Sub-sampling	634,065,068	58,816,336	9.28
Roof cover filtering	58,816,336	42,351,765	6.68
Segmentation	42,351,765	22,922,973	3.62

**Table 2:** Shape classification results for planar and non-planar segments.

Segment Class	Number of Segments	
	Planar	Non-Planar
Type-1	3208	117
Type-2	1757	264
Type-3	79	-

**Table 3:** Completeness of the models.

Region	Number of Beams	Method 1	Method 2	Method 3
Northern transept	199	61	129	150
Southern transept	194	53	117	146

## Data Availability

Not applicable.
